# Functional Outcomes and Successful Predictors of Lumbar Transforaminal Epidural Steroid Injections (LTFESIs) for Lumbar Radiculopathy Under Fluoroscopic Guidance: A Prospective Study

**DOI:** 10.7759/cureus.50257

**Published:** 2023-12-10

**Authors:** Karthikeyan Dhandapani, Debabrata Som, Prabhu Muthiahpandian, Andrew Miller, Aakaash Venkatesan, Mahak Baid, Naga Kishore Ausala, Raja Bhowmik, Mohammed Shabaz Faheem, Aditya Mani Subramani

**Affiliations:** 1 Orthopaedics and Traumatology, Aneurin Bevan University Health Board, Newport, GBR; 2 Orthopaedics and Traumatology, Mallareddy Institute of Medical Sciences, Hyderabad, IND; 3 Orthopedics and Traumatology, Jawaharlal Institute of Postgraduate Medical Education and Research, Pondicherry, IND; 4 Trauma and Orthopaedics, Grange University Hospital, Newport, GBR; 5 Surgery, Aneurin Bevan University Health Board, Newport, GBR

**Keywords:** intervertebral disc prolapse, economic burden, predictors of success, functional outcomes, transforaminal epidural steroid injection, lumbar radiculopathy

## Abstract

Background: Lumbar radiculopathy, a common and debilitating condition, often necessitates a multimodal approach for effective management. Lumbar transforaminal epidural steroid injection (LTFESI) has emerged as a valuable therapeutic option when conservative measures fall short. Recent interest in long-acting and non-particulate steroids prompts a critical examination of their impact on LTFESI outcomes. This prospective study aims to evaluate the efficacy of LTFESI in improving pain and functional outcomes in patients with lumbar radiculopathy, focusing on long-acting and non-particulate steroids, and analyse the associated economic burden.

Methods: The study, conducted from October 2017 to April 2019, involved 52 patients with lumbar radiculopathy meeting specific criteria. LTFESI was administered using a hospital-based prospective design. Functional outcomes were assessed using the Oswestry Disability Index (ODI) and Numeric Rating Scale (NRS) scores at various intervals. Statistical analyses were performed to identify predictors of successful outcomes.

Results: Participants (average age 43.22 years, 27 (51.92%) male) exhibited diverse Michigan State University (MSU) grade profiles and predominantly had pathology at the L4-5 level. The study demonstrated a significant and lasting functional improvement in 43 (82.69%) of patients after LTFESI. Patients with 2AB-type intervertebral disc prolapse (IVDP) showed lower response rates, emphasizing subtype influence. The efficacy of LTFESI was sustained for up to six months in almost 82.69% of patients, highlighting its potential for long-lasting benefits. The difference in the mean ODI score pre-injection and six months post-injection is statistically significant (p<0.0001). A total of four patients (7.69%) underwent surgical treatment for lumbar radiculopathy as their symptoms did not improve after injection. For all four patients (7.69%), surgery was done one month after injection. Five patients (9.61%) had ODI scores of more than 40, indicating severe disability at the end of six months. So, in nine patients (17.3%), the injection given was not effective at the end of six months, four (7.69%) of whom were operated on and five (9.61%) patients received conservative treatment. Thus, 43 (82.69%) of patients had a good outcome.

Discussion: The study reinforces LTFESI as an effective and safe intervention, providing substantial and lasting benefits for lumbar radiculopathy. The majority experienced immediate relief, supporting its role as an intermediate option between conservative management and surgery. Identified predictors of decreased success underscore the importance of early intervention and tailored treatment plans. The study emphasizes LTFESI's diagnostic and therapeutic potential, with economic benefits and safety highlighted.

Conclusion: LTFESI emerges as a safe and effective intervention for lumbar radiculopathy, offering substantial and enduring pain relief. The study contributes valuable insights into the nuanced outcomes of LTFESI, including the impact of IVDP subtypes, factors influencing success, and the procedure's cost-effectiveness. While acknowledging limitations, this work adds to the growing evidence supporting LTFESI as a crucial component in the management of lumbar radiculopathy.

## Introduction

Lumbar radiculopathy, characterised by radiating pain, sensory deficits, and motor weakness along the distribution of one or more lumbar spinal nerves, is a prevalent and debilitating condition affecting a significant portion of the global population [1]. The management of lumbar radiculopathy often involves a multimodal approach, with conservative measures such as physical therapy, analgesics, and lifestyle modifications serving as initial steps [2]. However, when conservative measures fail to provide adequate relief, lumbar transforaminal epidural steroid injection (LTFESI) emerges as a valuable therapeutic option [3].

LTFESI is a minimally invasive procedure that entails the targeted delivery of corticosteroids to the affected lumbar nerve roots, reducing inflammation and alleviating pain associated with lumbar radiculopathy [4]. Over the years, LTFESI has gained popularity due to its potential to provide substantial pain relief and enhance the functional status of patients, ultimately improving their quality of life [5].

In recent years, there has been a growing interest in the use of long-acting and non-particulate steroids in epidural injections, including LTFESI. Long-acting steroids, such as dexamethasone, have a prolonged duration of action, which may result in sustained pain relief and potentially reduce the need for repeated injections [6]. Non-particulate steroids, such as methylprednisolone acetate, have been proposed to have a lower risk of particulate-induced complications, such as embolism or neurotoxicity [7]. Despite their potential advantages, there is a paucity of research investigating the specific impact of these steroid formulations on the outcomes of LTFESI.

This study aims to address these critical knowledge gaps by comprehensively evaluating the efficacy of LTFESI in improving pain and functional outcomes in patients with lumbar radiculopathy, with a particular focus on the use of long-acting and non-particulate steroids. We will investigate whether these steroid formulations offer advantages in pain relief, functional improvement, and duration of effect. Moreover, we will seek to identify predictors of successful outcomes, helping clinicians make informed decisions regarding the choice of steroid formulation for individual patients.

Furthermore, this study will analyse the economic burden associated with LTFESI using different steroid formulations, shedding light on the cost-effectiveness of these interventions compared to other treatment modalities. By assessing the long-term cost implications of LTFESI with various steroids, we aim to provide valuable insights into healthcare resource allocation and optimisation.

By bridging the existing gaps in our understanding of LTFESI, with a specific focus on long-acting and non-particulate steroids, this study endeavours to enhance the evidence base for the management of lumbar radiculopathy, ultimately leading to improved patient care, prolonged pain relief, and cost-effective healthcare delivery.

## Materials and methods

This study was conducted in the Department of Orthopaedics, Malla Reddy Institute of Medical Sciences and Hospital, Hyderabad, India. The institute approved this study (127-46140-171-212654).

The main objective of this study was to evaluate the efficacy of LTFESI in improving pain and functional outcomes in patients with lumbar radiculopathy, and the study was conducted over a period spanning from October 2017 to April 2019, encompassing patients from all units within the Orthopedics Department. Data were collected at multiple follow-up intervals, including 24 hours, one month, three months, and six months, through a combination of verbal history and the review of indoor case sheets. Each patient's information was recorded using a standardised proforma. A total of 52 admitted patients were included in the study, with the sample size determined using Daniel's sample size formulae, taking into account an expected proportion of 0.442, a precision of 13%, and a desired confidence level of 95%. This choice was substantiated by prior literature, particularly Cooper et al.'s 2004 study, which reported a 44.2% positive therapy response at three months [8]. Inclusion criteria comprised patients aged 18 to 65 with lumbar radiculopathy confirmed by MRI and X-ray, single-level involvement of Intervertebral disc prolapse (IVDP), unsuccessful conservative treatment for three months, and an Oswestry Disability Index (ODI) score exceeding 40%. Exclusion criteria encompassed cases with more than two levels of IVDP, cauda equina syndrome, prior lumbar surgery, coagulation disorders, and uncontrolled type 2 diabetes mellitus. This study adopted a hospital-based prospective design to investigate the effectiveness of therapy for lumbar radiculopathy.

The technique employed in this procedure involves the following steps. First, after precisely determining the desired anatomical level, a local anaesthetic is administered after the test dose, and a brief waiting period is observed to allow for its effect. The patient is positioned prone on a surgical table compatible with fluoroscopy, adhering to strict sterile aseptic protocols. The back area from one vertebral space above to one below the intended injection site is meticulously cleansed and draped. Subsequently, a 23-gauge spinal needle is inserted and carefully advanced using fluoroscopy guidance until it contacts the intersection of the superior articular process and the lower border of the superior transverse process. After a slight withdrawal of the needle by 2 to 3 mL, it is directed towards the base of the appropriate pedicle, with slow and careful advancement, continuously monitored by fluoroscopy. The needle's position is verified using the fluoroscope's lateral projection before switching to the anteroposterior view. Ensuring that the needle is within the safe triangle, defined by the pedicle, exiting nerve root, and vertebral body, is crucial. Access to the S1 level is achieved with a 23-gauge spinal needle guided by fluoroscopy to the upper outer quadrant of the ipsilateral S1 foramen. Slow and controlled injection of 1 mL of nonionic contrast agent iohexol is administered, facilitating the visualisation of a perineurosheathogram. Similar injections are performed at each nerve root level, guided by C-arm fluoroscopy. If the desired contrast flow is not achieved, needle repositioning is carried out. Once proper contrast flow is confirmed, a mixture of 2 mL of dexamethasone (each millilitre containing 4 mg) and 2 mL of 0.25% bupivacaine is administered, and the patient is positioned supine for 10 minutes. Throughout the procedure, patient vitals were monitored.

Assessment and follow-up procedures involved advising patients against the use of opioids or NSAIDs during the trial period, with continuous monitoring for neurologic impairment, worsening pain, and the emergence of new pain symptoms. Initial assessments of ODI and Numeric Rating Scale (NRS) scores were performed at the outpatient department (OPD) before the injection, followed by evaluations at 24 hours, one month, three months, and six months post-injection. Data collection was carried out using a study-specific proforma. Treatment outcomes were determined based on the ODI and NRS scores, with success defined as a significant reduction in disability (ODI < 40%) and a pain reduction of over 50% on the NRS (rated 1, 2, and 3) at least one month after therapy. In terms of statistical analysis, data were entered into Microsoft Excel (Microsoft Corporation, Redmond, Washington), and EPI Info^TM^ (Version 7.2.2.2, Centers for Disease Control and Prevention (CDC), Atlanta) was used for assessment. Prevalence rates for various factors were expressed as percentages, continuous data were analysed using mean and standard deviation, categorical variables were assessed with the chi-square test, and comparisons of mean scores before, after, and at different injection intervals were conducted using Student's t-test, with statistical significance set at P < 0.0001.

## Results

From October 2017 to April 2019, the Department of Orthopaedics at Malla Reddy Institute of Medical Sciences and Hospital administered lumbar transforaminal epidural steroid injections to 52 patients in the operating room. IVDP at a single level affected 52 patients. After passing the inclusion and exclusion criteria, 52 patients were added to the research. Participants in the study had an average age of 43.22 ± 9.97 years. There were 27 men (51.92%) and 25 women (48.07%) in the study population.

**Figure 1:**
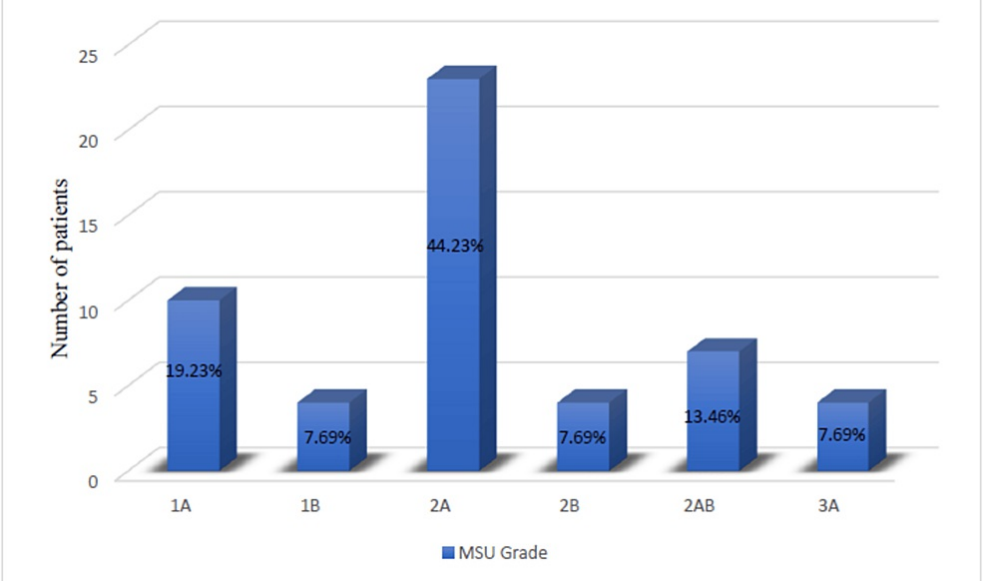
Distribution of Michigan State University grades

The Michigan State University (MSU) grade profile of the IVDP patients, based on the results of their lumbar spine MRIs, showed that all patients with IVDP received an MSU grade. Ten patients (19.23%) had type 1A, four (9.61%) had type 1B, 23 (44.23%) had type 2A, four (9.61%) had type 2B, seven (13.46%) had type 2AB, and four (9.61%) had type 3A. A graph showing this distribution is displayed in Figure 1.

For the level of pathology, there were 10 (19.2%) patients with L3-4, 31 (59.6%) patients with L4-5, and 11 (21.1%) patients with L5-S1 level disorders. In the study group, L4-5 pathology affected 31 (59.6%) of the population overall, followed by L5-S1 pathology 11 (21.1%) and L3-4 pathology 10 (19.2%).

ODI was calculated pre-injection, 24 hours, one month, three months, and six months after injection. Patients were scored, and the average scores are displayed in the table below, which showed a significant decrease in ODI mean of 56.61 pre-injection scores to 25.5 at six months post-injection (Table 1). The differences in the mean ODI score pre-injection, 24 hours, one month, three months, and six months after injection were statistically significant (p = <0.0001). This shows that the efficacy of ESI has lasted, on average, effectively six months in almost 43 (82.69%) patients, improving their pain as well as functional outcomes.

**Table 1:** Averages of the Oswestry Disability Index (ODI) with standard deviation and statistical significance at regular intervals A successful outcome was defined as a significant reduction of disability with an ODI score of less than 40%. The difference in the mean ODI score pre-operative and 24 hours after injection was statistically significant (p < 0.0001). The difference in the mean ODI score 24 hours and one month after injection was statistically significant (p < 0.0001). The difference in the mean ODI score one month post-injection and three months after injection was statistically significant (p < 0.0001). The difference in the mean ODI score three months post-injection and six months after injection was statistically significant (p = <0.0001).

	ODI (Mean)	Standard Deviation (SD)	P Value
Pre-Injection (n=52)	56.61	8.97	<0.0001
24 hours post (n=52)	45.84	9.11	<0.0001
1 month (n=52)	30.69	11.03	<0.0001
3 months (n=48)	26.83	8.76	<0.0001
6 months (n=48)	25.5	10.40	<0.0001

**Figure 2:**
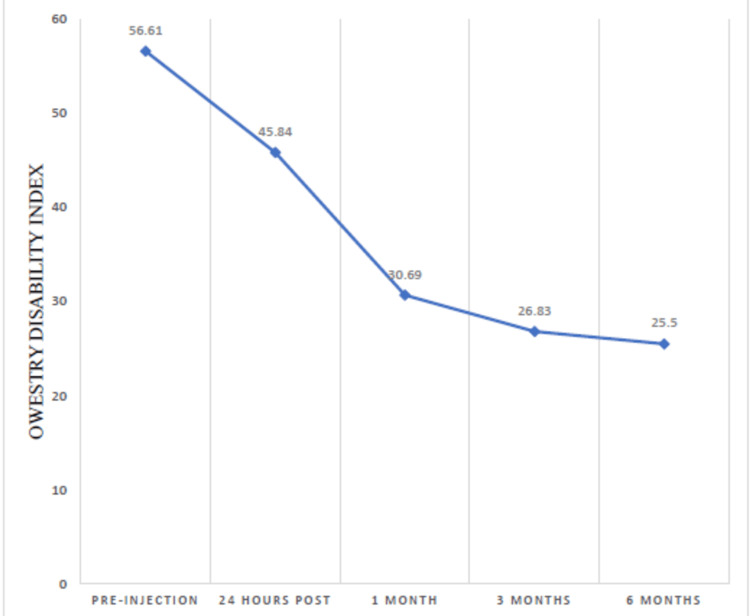
The trend of Oswestry Disability Index scores depicting a steady decline over time

The mean ODI score, calculated pre-injection and 24 hours, one month, three months, and six months after injection, showed a downward trend over time (Figure 2).

The number of patients who underwent surgery and the patients' ODI scores in relation to their MSU scores were calculated and showed that, in our study, IVDP was categorised using the MSU system. Interestingly, 2A was the most prevalent type in our sample, and functional outcomes stayed the same across all types. However, patients with 2AB-type IVDP demonstrated a lower response rate, with 2 (3.8%) out of 4 (7.69%) eventually requiring surgery. This observation highlights the potential influence of the IVDP subtype on treatment outcomes (Table 2).

**Table 2:** Oswestry Disability Index (ODI) averages related to Michigan State University grades and operated patients

	ODI, Pre-injection	ODI, 24 Hours Post-injection	ODI, 1 Month	ODI, 3 Months	ODI, 6 Months	Operated
1A	52	42	24.4	21	22.6	-
1B	58.5	45.5	30.5	23	19.5	-
2A	56.08	44.69	29.21	44.12	22.18	1
2B	61	52	35.5	24.66	29.33	1
2AB	60	50.28	39.14	36.8	35.2	2
3A	59	48.5	35.5	29	29.5	-

**Figure 3:**
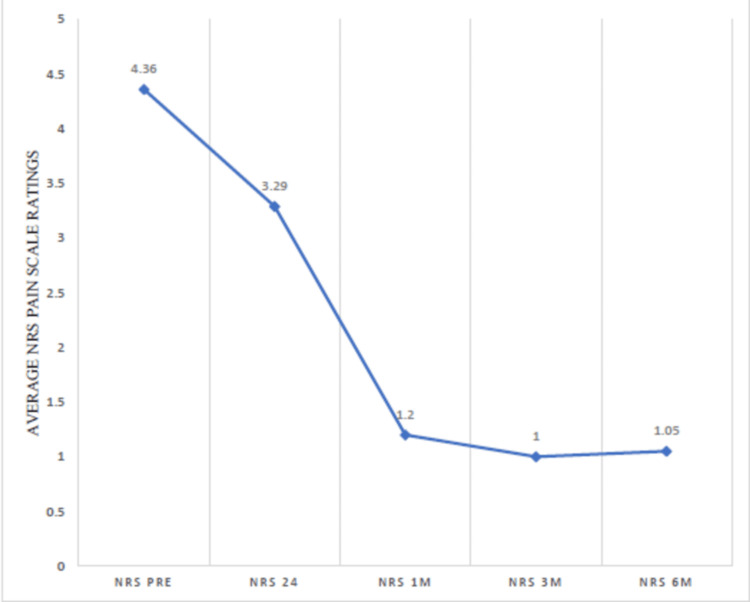
Numeric Rating Scale (NRS) ratings showing a downward trend after giving the epidural steroid injection

The NRS was used to calculate pain scores. Leg, buttock, and back pain levels were assessed independently in each of the three postures: standing, sitting, and squatting. NRS pain ratings at pre-injection, 24 hours, one month, three months, and six months after injection were calculated, which showed a downward trend of NRS pain ratings after giving the epidural steroid injection, from 4.36 at pre-injection score to 1.05 at six months post-injection (Figure 3).

Pain scores were rated separately for the leg, buttock, and back in three different postures: standing, sitting, and squatting positions. The NRS score trend showed a decreasing trend in each individual segment, and at six months, a median score of 1.5 was achieved in back squatting and 1.0 in all other positions, for example, back standing, back sitting, buttock squatting, buttock standing, buttock sitting, leg squatting, leg standing, and leg sitting. This demonstrated significant improvement in pain scores (Table 3).

**Table 3:** Numeric Rating Scale (NRS) pain ratings at one, three, and six months after injection IQR: interquartile range.

Study Participant Status	NRS Rating Median (IQR) at 1 Month	NRS Rating Median (IQR) at 3 Months	NRS Rating Median (IQR) at 6 Months
Back – Squatting	2.00(1,2)	1.00(1,2)	1.5(1,3)
Back – Standing	1.00(1,2)	1.00(1,1)	1.00(1,2)
Back – Sitting	2.00(1,2)	1.00(1,1)	1.00(1,2)
Buttock – Squatting	1.00(1,1)	1.00(1,1)	1.00(0,1.5)
Buttock – Standing	1.00(1,1)	1.00(0,1)	1.00(0,1)
Buttock – Sitting	1.00(1,2)	1.00(0,1)	1.00(0,1)
Leg – Squatting	1.00(1,2)	1.00(1,2)	1.00(0,2.5)
Leg – Standing	1.00(1,2)	1.00(0,1.5)	1.00(0,2)
Leg – Sitting	1.00(1,2)	1.00(0,2)	1.00(0,2)

Four patients (7.69%) in total underwent surgery to address their lumbar radiculopathy, as their symptoms did not improve after injection. Surgery was performed on all four (7.69%) individuals a month following their injections. At the end of six months, five patients (9.61%) had ODI scores greater than 40, indicating severe disability. Thus, after six months, the injection was ineffective in nine patients (17.3%), five (9.6%) of whom were still receiving conservative care, and four (7.69%) of whom underwent surgery. Consequently, 43 (82.69%) of the patients achieved satisfactory outcomes.

## Discussion

The results of this prospective trial, which examined the effectiveness of LTFESI in patients with single-level IVDP, offer important new perspectives on how to treat lumbar radiculopathy.

The participants' average age in the study was 43.22 years, reflecting that lumbar radiculopathy can affect individuals across a wide age range. This diversity in age highlights the need for treatment options that cater to both younger and older patients with this condition. The almost equal gender distribution in the study (25 (48.07%) females and 27 (51.92%) males) underscores the non-discriminatory nature of lumbar radiculopathy.

The utilisation of the MSU grading system to classify IVDP based on lumbar spine MRI provides a comprehensive understanding of the spectrum of disease severity within the study cohort [9]. This classification allows for the differentiation of patients with varying levels of IVDP, which may have implications for treatment decisions and outcomes. In our study, IVDP was categorised using the MSU system.

Interestingly, 2A was the most prevalent type in our sample, and functional outcomes stayed the same across all types. However, patients with 2AB-type IVDP demonstrated a lower response rate, with 2 (3.8%) out of 4 (7.69%) eventually requiring surgery. This observation highlights the potential influence of the IVDP subtype on treatment outcomes.

The identification of L4-5 as the most common level of pathology, affecting nearly 60% of the total population, aligns with existing literature. L4-5 has been consistently reported as a frequent site of IVDP, which emphasises the importance of tailored interventions for this specific lumbar level.

Our study findings are consistent with those of Buttermann et al., who conducted a prospective randomised controlled study involving 169 patients [10]. In their study, 56% of patients who received epidural steroid injections for lumbar radiculopathy experienced a positive outcome. In contrast, our study found that 43 (82.69%) patients had successful functional results after LTFESI. Although our study was not a randomised controlled trial, it corroborates the effectiveness of LTFESI in reducing symptoms and disability.

One of the most significant findings of this study is the overall effectiveness of LTFESI. A substantial proportion of patients, 43 (82.69%), experienced excellent functional outcomes, as evidenced by significant improvements in the ODI scores. These findings support LTFESI as a viable and valuable treatment option for patients suffering from lumbar radiculopathy. It serves as an intermediate option between conservative management and surgical intervention, potentially reducing the need for more invasive procedures in a majority of cases.

Our findings align with the North American Spine Society's recommendation that LTFESI provides substantial pain relief [11]. In our study, 43 (82.69%) of patients experienced significant immediate relief, a trend consistent with the literature. We noted that 33 (65%) of patients reported more than 50% pain relief for three months following treatment, further supporting the efficacy of LTFESI.

While the majority of patients benefited from LTFESI, it is noteworthy that a small percentage, 9 (17.3%), did not achieve the desired relief. Among these patients, 4 (7.69%) required surgical intervention, suggesting that a subgroup of individuals may have conditions that are less responsive to epidural steroid injections. The study's identification of this subset emphasises the importance of individualised treatment plans and close monitoring for patients who do not respond optimally to LTFESI.

The statistical analysis revealed that the efficacy of LTFESI was sustained for up to six months in almost 43 (82.69%) patients. This is a noteworthy discovery because it implies that even while the steroid impact may fade over time as the disease process advances, a sizable proportion of patients may still achieve long-lasting pain reduction and functional improvement. This highlights the potential for LTFESI to provide substantial and lasting benefits to those suffering from lumbar radiculopathy. The presumed mechanisms of action include the anti-nociceptive qualities of steroids and anaesthetics, nerve membrane stabilisation, the "washout" effect reducing inflammation mediators, and the potent anti-inflammatory nature of steroids.

Our study identified factors associated with decreased success, including spinal instability, disc herniation progression, and symptom duration exceeding one year. These findings underscore the importance of early intervention and suggest that chronic neural compression may reduce the efficacy of steroid treatment.

A considerable number of patients with disc herniation got epidural steroid injections in a meta-analysis by Lavalle et al., and a small number later required discectomy. Our study also observed that 4 (7.69%) of patients with IVDP underwent discectomy after LTFESI, with a similar time frame between injection and surgery [12].

Our study highlights the diagnostic and therapeutic potential of LTFESI, in addition to its therapeutic role. LTFESI assists in both diagnosing and treating the underlying problem by precisely delivering medication to the pathogenic spot. Its clinical usefulness in treating lumbar radiculopathy is improved by this dual function.

The study reported only minor complications, such as transient numbness, with no significant adverse events. This reaffirms the safety of LTFESI as a minimally invasive procedure. The low incidence of complications further supports its role as a valuable treatment option with a favourable risk-benefit profile. Our study highlights the safety of the fluoroscopic transforaminal approach, which avoided serious complications such as severe bleeding or dural puncture, which are frequently related to blind epidural injection methods. This supports the case for routine fluoroscopy use to improve safety and accuracy.

Our study suggests that LTFESI can provide economic benefits by reducing the burden on patients and healthcare systems. Effective pain relief and a decreased need for surgery contribute to cost savings and improved resource allocation.

The use of fluoroscopy in LTFESI is paramount, enabling the precise and accurate delivery of medication to the point where the inflamed nerve root meets the herniated nucleus pulposus (HNP). If there is no fluoroscopic guidance, then there is a considerable risk of misplacing needle into unintended areas [13]. Furthermore, this technique relies on normal epidural anatomy, which is not guaranteed in all cases. The site-specific modification necessitated by abnormal anatomy would be impossible without fluoroscopy, emphasising its critical role in the procedure [14].

Several factors can influence the success of LTFESI. We observed that lower success rates were related to spinal instability, greater disc herniation, and symptom duration of more than a year [15]. This underscores the importance of early intervention and tailored treatment plans. Additionally, irreversible changes related to cases with prolonged symptom duration chronic neural compression may make the nerve root less sensitive to steroid treatment.

This research has certain critical limitations. First, it lacks the structure of a randomised controlled study, which can impact its validity. Secondly, the study faced challenges arising from the relatively small size of the study population, particularly when evaluating outcomes in connection with the MSU classification for disc prolapse [16]. Moreover, the follow-up duration in this study was of a midterm nature, highlighting the need for an extended, long-term follow-up period to thoroughly evaluate the sustained effects of the intervention. It is crucial to recognise the study's shortcomings, such as its small sample size and short midterm follow-up period. These constraints may impact the generalizability of the findings and limit the ability to assess long-term outcomes. Future research with larger patient cohorts and extended follow-up periods could provide a more comprehensive understanding of the sustained effects of LTFESI.

## Conclusions

This transforaminal epidural steroid therapy exhibits superior results in ODI ratings and NRS scores. LTFESI leads to substantial disability improvement, with the most significant benefits realised within one month, although further improvements taper off, with sustained relief observed in a majority of patients lasting beyond six months. LTFESI also enhances patient satisfaction and provides enduring pain relief for most patients, offering a cost-effective treatment option. Furthermore, patients with the 2AB-type classification of the MSU classification of IVDP were more likely to require surgery and experience adverse outcomes, although the study's limited population size somewhat constrained the precise delineation of the association between various types of MSU classification of IVDP. Patients receiving LTFESI within one year of symptom onset reported superior outcomes and reduced reliance on oral analgesics. Lumbar Transforaminal Epidural Steroid Injections serve a dual purpose, offering both diagnostic and therapeutic benefits in patient management.

Moreover, the study highlights the cost-effectiveness of LTFESI and its potential to reduce the reliance on surgical interventions. While further research is needed to refine our understanding of LTFESI's long-term benefits and its applicability to different IVDP subtypes, our study adds valuable insights to the field of interventional pain management. The majority of patients experienced significant and lasting benefits from LTFESI, making it a valuable intermediate treatment option between conservative management and surgery. While a small subset of patients did not achieve optimal relief, the safety profile of LTFESI and its potential for prolonged efficacy make it a valuable component of the treatment approach for lumbar radiculopathy.
